# Cobaltabis(Dicarbollide) [*o*-COSAN]^−^ for Boron Neutron Capture Therapy of Head and Neck Cancer: Biodistribution and Irradiation Studies in an Experimental Oral Cancer Model

**DOI:** 10.3390/ph17101367

**Published:** 2024-10-14

**Authors:** Mónica A. Palmieri, Andrea Monti Hughes, Verónica A. Trivillin, Marcela A. Garabalino, Paula S. Ramos, Silvia I. Thorp, Paula Curotto, Emiliano C. C. Pozzi, Miquel Nuez Martínez, Francesc Teixidor, Clara Viñas, Amanda E. Schwint

**Affiliations:** 1 Departamento de Biodiversidad y Biología Experimental (DBBE), Facultad de Ciencias Exactas y Naturales (FCEN)-Universidad de Buenos Aires (UBA), Ciudad Autónoma de Buenos Aires C1428EGA, Argentina; 2 División Patología de la Radiación, Departamento de Radiobiología, Gerencia Química Nuclear y Ciencias de la Salud, Gerencia de Área Aplicaciones Nucleares a la Salud (GAANS), Comisión Nacional de Energía Atómica (CNEA), Buenos Aires B1650KNA, Argentina; trivilli@cnea.gov.ar (V.A.T.); garabalino@cnea.gov.ar (M.A.G.); paula.sofi.ramos@gmail.com (P.S.R.); mandyschwint@gmail.com (A.E.S.); 3 Consejo Nacional de Investigaciones Científicas y Técnicas (CONICET), Ciudad Autónoma de Buenos Aires B1425FQB, Argentina; 4 Sub-Gerencia Instrumentación y Control, Gerencia de Área Producción de Radioisótopos y Aplicaciones de la Radiación (GAPRyAR), Centro Atómico Ezeiza (CAE), Comisión Nacional de Energía Atómica (CNEA), Buenos Aires B1802AYA, Argentina; silviathorp@cnea.gob.ar; 5 Departamento de Reactores de Investigación y Producción, Gerencia de Área Producción de Radioisótopos y Aplicaciones de la Radiación (GAPRyAR), Centro Atómico Ezeiza (CAE), Comisión Nacional de Energía Atómica (CNEA), Buenos Aires B1802AYA, Argentina; curotto@cae.cnea.gov.ar (P.C.); epozzi@cnea.gob.ar (E.C.C.P.); 6 Institut de Ciència de Materials de Barcelona (ICMAB)-Consejo Superior de Investigaciones Científicas (CSIC), 08193 Bellaterra, Spain; mnuez@icmab.es (M.N.M.); teixidor@icmab.es (F.T.); clara@icmab.es (C.V.)

**Keywords:** boron carrier, metallacarborane, Na[*o*-COSAN], biodistribution studies, boron neutron capture therapy, BNCT, head and neck cancer, hamster

## Abstract

Background: Boron neutron capture therapy (BNCT) is a tumor-selective particle radiotherapy that combines preferential boron accumulation in tumors and neutron irradiation. Based on previous studies in tumor-bearing mice, this study evaluated the biodistribution of the sodium salt of cobaltabis(dicarbollide) (Na[3,3′-Co(C_2_B_9_H_11_)_2_], abbreviated as Na[*o*-COSAN]) in the hamster cheek pouch oral cancer model and the Na[*o*-COSAN]/BNCT therapeutic effect on tumors and induced radiotoxicity. The synthesis and comprehensive characterization of ^10^B-enriched trimethylammonium salt of *nido*-[7,8-C_2_^10^B_9_H_12_]^−^*o*-carborane, along with the cesium and sodium salts of [*o*-^10^COSAN] cobaltabis(dicarbollide) are reported here for the first time. Methods: Hamsters bearing tumors were injected with Na[*o*-COSAN] (7.5 mg B/kg) and euthanized at different time-points after injection (30 min, 2, 3, 5, and 18 h post-administration) to evaluate boron uptake in different tissues/organs. Based on these results, tumor-bearing animals were treated with Na[^10^B-*o*-COSAN]/BNCT (7.5 mg B/kg b.w., 3 h), prescribing 5 Gy total in absorbed dose to the precancerous tissue surrounding tumors, i.e., the dose-limiting tissue. Results: Na[*o*-^10^COSAN] exhibited no toxicity. Although biodistribution studies employing Na[*o*-COSAN] have shown low absolute boron concentration in the tumor (approx. 11 ppm), Na[*o*-^10^COSAN]/BNCT induced a high and significant therapeutic effect on tumors versus the control group (cancerized, untreated animals). Moreover, only half of the animals exhibited severe mucositis in the precancerous dose-limiting tissue after BNCT, which resolved completely at 21 days after irradiation. Conclusions: Na[*o*-^10^COSAN] would be potentially useful to treat head and neck cancer with BNCT.

## 1. Introduction

Worldwide, head and neck tumors rank sixth in the list of most common cancers. A total of 90% of these are squamous cell carcinomas of the oral cavity, pharynx, and larynx. The major risk factors are tobacco smoking and/or alcohol drinking, oncogenic viruses, and lifestyle like dietary factors, oral hygiene, etc. [1,2,3]. These tumors are not an easy target for anticancer strategies. Generally, they require an aggressive and multidisciplinary approach, depending on tumor location and histological characteristics. They are highly heterogeneous. Surgery, radio/chemotherapy, molecular inhibitors, and immunotherapy are used [4,5,6,7,8]. However, survival and quality of life have not been improved significantlydue to radioresistance, drug-resistance acquisition over time, and significant toxicities. In this sense, more effective and tolerable strategies are urgently needed [2].

Boron neutron capture therapy (BNCT) has proved particularly effective for the treatment of head and neck malignancies [9,10,11]. BNCT is a particle-selective radiotherapy that consists of two components: a boron compound that preferentially accumulates in tumor cells followed by a neutron irradiation. After ^10^B captures a thermal neutron, two high LET α and Li particles are released and travel a short distance around the diameter of one cell. In this way, BNCT is a biological targeting therapy, killing those boron-loaded tumor cells while preserving surrounding normal cells [12].

Two boron agents have been widely used for clinical BNCT trials for different pathologies: boronphenylalanine (BPA) and sodium borocaptate (BSH). Clinical results have been very promising regarding tumor response, patient survival, and quality of life [13,14,15,16,17,18,19,20,21]. Multiple efforts were made to optimize the use of^10^B compounds currently authorized for use in humans and to bridge the gap between research and clinical application [22,23,24].

Animal models are essential for studying the potential therapeutic effect of a new boron compound for BNCT and induced toxicities. Within the context of head and neck cancer research, the hamster cheek pouch has been widely described as one of the most ideal animal models to study oral cancer development, anti-cancer strategies, and associated toxicity, particularly mucositis [25,26,27]. The carcinogenesis protocol used to induce tumors consists in the topical application of subthreshold doses of the complete carcinogen DMBA (7,12-dimethylbenz[a]anthracene), simulating the action of smoking in humans [28,29]. This protocol induces tumors surrounded by a precancerous tissue (the dose-limiting tissue) from which additional tumors arise and where mucositis develops after treatment [29]. Our group has extensively studied the radiobiology of BNCT in this experimental oral cancer model. We have evaluated strategies to increase the therapeutic effect of BNCT mediated by those boron compounds approved for use in humans, evaluated new boron carriers, and studied mucositis induced by BNCT [29,30,31].

Both BPA and BSH have been widely used in BNCT for head and neck patients. However, it is reported that they have limitations related to boron loading, solubility, tumor cell targeting, and accumulation in tumor and healthy tissue. This poses the need for new boron compounds that could overcome these restrictions, i.e., being more selective, nontoxic, and effective in boron delivery [32,33,34]. It is known that boron forms molecules by covalent self-bonding as carbon. These polyhedral compounds are named boranes and heteroboranes. One of the most commonly used in the medicinal chemistry field is the icosahedral dicarba-*closo*-dodecaboranes, whose formulas are *closo*1,2-C_2_B_10_H_12_, 1,7-C_2_B_10_H_12_ and 1,12-C_2_B_10_H_12_ for *ortho*-, *meta*- and *para*- isomers, respectively [35]. Furthermore, these icosahedral carboranes, composed of C and B atoms, can incorporate metals into their icosahedral clusters, forming metallacarboranes [35]. This transformation allows these small molecules to serve as potential multimodal agents, offering both diagnostic and multiple therapeutic functionalities simultaneously [36]. In 2014, Tarrés et al. [37] reported on the synthesis of Na[*o*-COSAN] (Na[*o*-COSAN]) in terms of the cellular viability, uptake, and intracellular distribution in different cell lines and biodistribution studies in mice, using a single dose (7.5 mg B/kg). They demonstrated high boron content, low toxicity, high uptake by cancer cells, nucleus accumulation *in vitro*, and its capacity to strongly interact with DNA and proteins [38]. Moreover, Na[*o*-COSAN] was shown to be a good candidate for dual cancer treatment for glioblastoma, i.e., chemotherapy action and BNCT boron carrier [39]. In this sense, the aim of the present study was to evaluate, for the first time, the biodistribution of Na[*o*-^10^COSAN] in the hamster cheek pouch oral cancer model and assess the therapeutic effect and radiotoxicity induced by BNCT mediated by Na[*o*-^10^COSAN] in this experimental animal model. This study will contribute to the optimization of BNCT, particularly for the treatment of head and neck cancer.

## 2. Results

### 2.1. Synthesis of ^10^B-Enriched Cesium and Sodium Salts of [o-^10^COSAN]^−^

To increase the ^10^B content in the cobaltabisdicarbollide cluster for use in boron neutron capture therapy targeting head and neck cancer in an experimental oral cancer model, the synthesis of Na[*o*-^10^COSAN] was carried out using established [40,41] methods but starting from 1,2-closo-C_2_^10^B_10_H_12_. The first step involved synthesizing [HNMe_3_][7,8-*nido*-C_2_^10^B_9_H_12_], which served as a ligand in the subsequent complexation reaction. The [*o*-^10^COSAN]^−^ complex was initially isolated as salt of Cs^+^, which is water-insoluble and achieved a maximum yield of 74.5%. The water-soluble Na^+^, suitable for BNCT irradiation, was then prepared from the Cs^+^ salt through cation exchange chromatography, resulting in a yield of 96%. Characterization revealed clear differences in the IR spectra of Cs[*o*-^10^COSAN] and Na[*o*-^10^COSAN] in the range 3600–3200 cm^−1^ (see Figure S19 at the Supplementary Materials), consistent with their distinct water-solubility properties. Additionally, the IR spectra of natural boron Na[*o*-COSAN] and ^10^B-enriched Na[*o*-^10^COSAN] exhibit a 10 cm^−1^ blueshift around 2550 cm^−1^*,* corresponding to B-H bond vibrations [42]. This shift is attributed to the difference between the ν(^10^B-H) and ν(^11^B-H) stretching frequencies, as illustrated in Figure S20. The MALDI-TOF-MS spectra of the synthesized ^10^B-enriched Cs[*o*-^10^COSAN] at the anode showed two peaks at *m*/*z* 309.086 (intensity: 72,640.562) and at 310.090 (intensity: 9642.227), corresponding to pure ^10^B-enriched [*o*-^10^COSAN]^−^ and [*o*-^10^COSAN]^−^ containing ^11^B atoms, respectively. From this mass spectrum, a percentage of 99.35% ^10^B in the synthesized enriched [*o*-^10^COSAN]^−^ was calculated, as detailed in Figure S12 of the Supplementary Materials.

### 2.2. Biodistribution Studies

**Figure 1:**
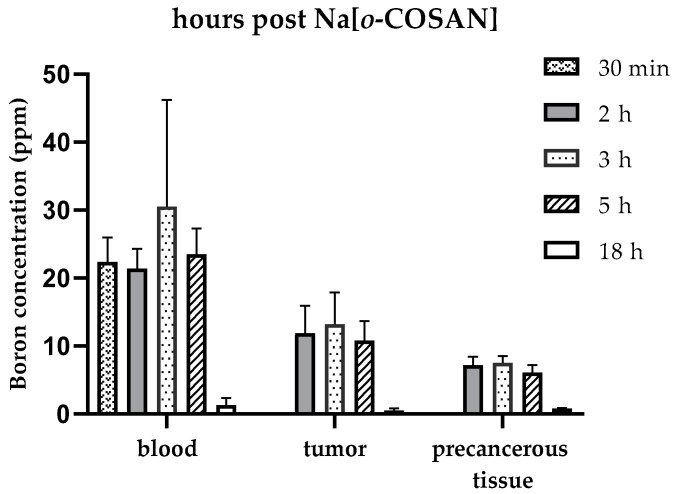
Boron concentration values (ppm) for blood, tumor, and precancerous tissue at different time-points post Na[*o*-COSAN] injection: 30 min, 2 h, 3 h, 5 h, and 18 h.

No short-term toxicity was observed when the sodium salt of natural boron [*o*-COSAN]^−^ was studied. Blood boron concentration values were similar and around 22 ppm at 30 min, 2 h, 3 h, and 5 h after Na[*o*-COSAN] injection. However, over the 2–5 h period, tumor boron concentration increased to a maximum of 13 ppm (Figure 1 and Table 1). The tumor/blood (T/B) ratio for times 2, 3 and 5 h was 0.6, 1.6 and 1.8 respectively, while the tumor/precancerous tissue (T/Pr) ratio for these three time-points was 1.6, 1.8 and 1.8 respectively (Table 2). Given that the tumor boron concentration values were below 20 ppm and all ratios (T/B and T/Pr) were below 3, the protocol would be not considered potentially therapeutic in terms of the traditional BNCT literature [32]. However, we decided to proceed with Na[*o*-COSAN]/BNCT radiobiological experiments based on our previous experience with the GB-10 boron compound [29]. Based on these studies, we performed Na[*o*-COSAN]/BNCT studies 3 h after Na[*o*-COSAN] injection, when tumor concentration was 11.0 ± 3.5 ppm (*n* = 5 tumors) and boron concentration in the dose-limiting tissue, the precancerous tissue surrounding tumors, was 7.5 ± 1.0 ppm (*n* = 3 animals) (Table 1).

### 2.3. BNCT In Vivo Studies

#### 2.3.1. Mucositis

The degree of mucositis was recorded weekly. At 7 days after BNCT, 50% of the hamsters exhibited moderate to severe mucositis in the precancerous tissue. Severe mucositis resolved rapidly and at 10 days after BNCT, all animals had slight to moderate mucositis. At 21 days after BNCT, all animals exhibited slight mucositis (Table 3).

#### 2.3.2. Therapeutic Effect of BNCT Mediated by Na[*o*-^10^COSAN]

At 28 days after treatment, BNCT mediated by Na[*o*-^10^COSAN] induced an 81% of overall tumor response (partial remission + complete remission), being significantly higher than the percentage of overall responses reported in the control group (21% due to spontaneous remissions; Table 4). Moreover, at the end of the follow up, 52% of the tumors responded completely to Na[*o*-^10^COSAN]/BNCT (versus 9% reported in the control group; Table 3, *p* < 0.02). Regarding those tumors that responded partially, 100% reduced their initial volumes by more than 50% (Table 4).

**Figure 2:**
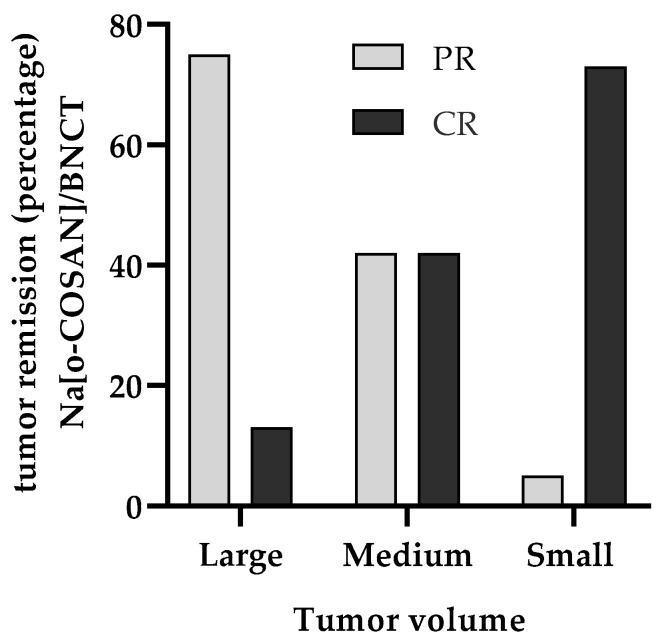
Therapeutic effect of Na[*o*-^10^COSAN]/BNCT. Percentage of tumors with partial remission (PR) and complete remission (CR) considering tumor volume at the time of irradiation: small (<10 mm^3^), medium (≥10 <100 mm^3^), and large (≥100 mm^3^).

When the tumors were classified by volume at the time of irradiation, it was also observed that one large tumor showed a complete remission and the rest had a reduction in volume of more than 50% of their initial volume. In the case of small- and medium-sized tumors, 73% and 42% of the tumors showed complete remissions (respectively) and the rest showed a reduction of more than 75% of their initial volume (Figure 2 and Table 5).

## 3. Discussion

In this study, we evaluated the potential use of Na[*o*-COSAN] as a boron carrier for BNCT for the treatment of head and neck tumors in the hamster cheek pouch experimental model of oral cancer. Although biodistribution studies employing Na[*o*-COSAN] have shown low absolute boron concentration in the tumor (about 11 ppm of ^10^B), BNCT mediated by Na[*o*-^10^COSAN] induced a high and significant therapeutic effect on tumors versus the control group (cancerized, untreated animals). Moreover, only half of the animals exhibited moderate to severe mucositis in the precancerous dose-limiting tissue after BNCT, which resolved completely at 21 days after irradiation.

The introduction of new boron compounds that could overcome BPA and BSH limitations is a need in BNCT. There are plenty of new boron compounds and an important step is to introduce them in the clinics [11,32,33,34]. Na[*o*-COSAN], compared for example to BPA, is easy to prepare for injection (no issues regarding solubility) and in this work, we demonstrated significant therapeutic results while reducing toxicity. BPA is a boron compound widely used in BNCT and has demonstrated significant therapeutic effect with reversible and manageable toxicities in head and neck clinical veterinary and human patients [9,11,44]. Our group has wide experience in BNCT mediated by BPA studies in the hamster cheek pouch oral cancer model. We demonstrated that BPA tumor accumulation is around the recommended values in the literature (20 ppm), and overall tumor responses are similar to those reported here for Na[*o*-^10^COSAN]/BNCT. However, when comparing toxicity, Na[*o*-^10^COSAN]/BNCT exhibited less toxicity in precancerous tissue vs BPA/BNCT [45]. With this in mind, Na[*o*-^10^COSAN] would improve BNCT for the treatment of head and neck cancer.

The efficiency of Na[*o*-COSAN] as a boron carrier for BNCT may be attributed to the ability of [*o*-COSAN]^−^ anions to cross both artificial bilayer membranes and cell membranes [37,38] through a flip-flop translocation mechanism, as demonstrated theoretically [46]. Once inside the cell’s cytoplasm, [*o*-COSAN]^−^ anions enter the cell’s nucleus [38]. This process is supported by the findings of Tagliazucchia et al. [47] who used theoretical methods to study the effect of charge and hydrophobicity on the translocation of model particles through the nuclear pore complex. They discovered that generic hydrophobicity and negative charge of nanoparticles are essential for their translocation. Once in the nucleus, [*o*-COSAN]^−^ anions interact with DNA in an anionic way. Consequently, following the uptake of [*o*-COSAN]^−^ anions, ^10^B is mainly localized within or near the nucleus. To emphasize that, *in silico* studies using Monte Carlo simulations have shown that energy deposition in the nucleus of cells exposed to neutron reactions results in a more efficient cell-killing effect compared to a uniform distribution throughout the entire cell. These results could suggest that, even at low concentrations, ^10^B from Na[*o*-COSAN] significantly enhances its value as a carrier-free drug for cancer treatment with BNCT.

Radiobiological studies in *in vitro* and *in vivo* models are necessary to select which boron compound would be interesting to introduce in a clinical scenario [23]. In the literature, several requirements have been stated regarding a potentially ideal boron delivery agent: boron absolute concentrations of 20–30 µg ^10^B/g in the tumor to reduce irradiation times and the associated background dose; high tumor/normal tissue boron concentration (T/N) and tumor/blood (T/B) ratios; low systemic toxicity; and rapid clearance from blood and normal tissues while persisting in tumor during neutron irradiation [32]. However, boron compounds generally do not fulfill all these requirements and previous studies have demonstrated that the requirements could be less stringent. For example, our group has shown that GB-10 (Na_2_[^10^B_10_H_10_]) exhibited mean absolute boron concentration values between 9.5 and 18 ppm in hamster cheek pouch tumors, with T/N and T/B ratios of 1 to 1.3 and 0.5 to 0.9 respectively [29,31]. However, we demonstrated a significant therapeutic effect of GB-10/BNCT vs. cancerized untreated animals: almost 50% (2.2 Gy tumor-absorbed dose) to 70% (7.92 Gy tumor-absorbed dose) overall tumor responses corresponding to Olaiz et al. [31] and Trivillin et al. [29], respectively. In these studies, no severe radiotoxic effects in precancerous tissue were observed. Another example came from Tsujino et al. [48], who studied a boron-conjugated 4-iodophenylbutanamide (BC-IP) for the treatment of brain cancer. They reported that although its accumulation in tumors was low, BC-IP/BNCT significantly enhanced the survival of glioma-bearing rats compared to untreated animals and those treated with neutrons only. The present study demonstrated that although the animals injected with Na[*o*-COSAN] (7.5 mg ^10^B/kg b.w., 3 h post injection) exhibited low tumor boron absolute uptake and T/B and T/N ratios, a high and significant therapeutic effect of Na[*o*-^10^COSAN]/BNCT on tumors was evidenced while preserving the precancerous dose-limiting tissue.

The present results are of significance in BNCT. However, there is room for improvement. For example, increasing tumor complete responses of large tumors after Na[*o*-^10^COSAN]/BNCT would be an asset, as unresectable large tumors significantly limit the treatment, survival, and quality of life [44]. In previous studies we demonstrated that the electroporation technique improved boron uptake and thus increased tumor control and complete responses employing the boron compound GB-10. This technique is based on the local application of an electric field to the tumor which permeabilizes the cell membranes, increasing the passage of molecules into the cytosol [31]. With this in mind, future studies will be aimed at combining Na[*o*-^10^COSAN]/BNCT with electroporation to enhance tumor boron uptake, T/B and T/Pr ratios, and increase tumor control, particularly of large tumors.

Na[*o*-^10^COSAN]/BNCT induced moderate to severe mucositis in 50% of animals, indicating potential toxicity. However, this mucositis was reversible. In this study, chronic effects, late-onset toxicities, and overall survival benefits could not be assessed due to the negative effects of the cancerization protocol employed on the clinical status of the animals. For that aim, future studies will be performed employing cancerization protocols that allow for medium- and long-term studies like the 8-week cancerization protocol (3-month follow-up) or 6-week protocol (8-month follow-up) [49]. Moreover, the evaluation of Na[*o*-^10^COSAN]/BNCT combined with strategies that could mitigate radiotoxic effects like radioprotectors will be considered. Our group has experience in employing radioprotectors to mitigate the mucositis or dermatitis induced by BNCT [50]. Another potential strategy that could help to reduce BNCT-induced toxicity could be the combined administration of boron compounds with different characteristics or to adjust the dose delivered and perform a double BNCT, for example. Lowering the dose in each irradiation would allow for lower toxicity after each application, but higher doses to the tumor in total.

Another possible step to improve the Na[*o*-COSAN]/BNCT therapeutic effect could be to increase the Na[*o*-COSAN] injected dose in the animals. However, Fuentes et al. [38] have reported a limitation associated to Na[*o*-COSAN], i.e., the formation of aggregates that accumulate mostly in the lung. This should be studied in detail, regarding the effect of these aggregates in the lung and their potential negative effects in the animals. Meanwhile, an alternative route of administration of the compound would be topical administration to the tumor. This would avoid the systemic exposure to Na[*o*-COSAN] of the organs, particularly lungs. An alternative effective strategy to injecting higher Na[*o*-COSAN] doses to the animals could be to test a Double Na[*o*-^10^COSAN]/BNCT. We have reported this protocol previously (employing as boron compounds BPA, GB-10 and liposomes) showing a significant increase in BNCT therapeutic effect without additional costs in terms of radiotoxicity in the precancerous dose-limiting tissue [30]. Based on the results obtained in the present study, as severe and moderate mucositis resolved at 14 days after BNCT, we propose that a second BNCT full-dose irradiation to precancerous tissue could be applied at 14 days after the first BNCT. This would allow the treatment of those tumor cell populations that were refractory to the first treatment, thus improving overall responses and complete tumor remissions. Moreover, the combination of Na[*o*-COSAN] with electroporation, in keeping with Olaiz et al. [31] employing the boron compound GB-10, could increase tumor boron uptake and T/B and T/N ratios, thus enhancing BNCT tumor control.

Herein we demonstrated that Na[*o*-COSAN] could be a potential boron compound to be introduced for the treatment of head and neck cancer patients with BNCT. However, a detailed study regarding its toxicity has to be performed first in small animal models, to then assess the need to move on to large animal studies (depending on the models available and issues to be addressed) and then introduce it in clinical veterinary scenarios and finally humans. Particularly, comparative veterinary oncology could be an interesting step before humans, as pets provide valuable, predictive, translational results for human cancer patients (they share similar environments, immune systems, sizes, etc.), while providing a cutting-edge therapy for them [51].

In conclusion, we demonstrated, for the first time, the therapeutic effect with reversible radiotoxicity of BNCT mediated by Na[*o*-^10^COSAN] in the hamster oral cancer model. This study contributes to the optimization of BNCT and development of new boron compounds, particularly for BNCT of head and neck cancer.

## 4. Materials and Methods

### 4.1. Animal Model

Syrian hamsters 6 to 8 weeks old were exposed to the optimized classical carcinogenesis protocol, which consists in the topical application of DMBA in mineral oil (0.5%) in the right cheek pouch twice a week for 12 weeks, with two interruptions (fourth and fifth application) completed at the end of the protocol [45]. Hamsters bearing tumors were assigned to Na[*o*-COSAN] biodistribution studies. Based on these results, another set of hamsters were then treated with BNCT mediated by Na[*o*-COSAN]. A control group of cancerized untreated animals was also included.

The experiments described below were designed based on the 3Rs (replacement, refinement, and reduction principles) and considering the guidelines published by the National Institute of Health in the USA regarding the care and use of laboratory animals in its eight edition from 2011. All protocols were previously approved by the Argentine National Atomic Energy Commission Animal Care and Use Committee (CICUAL-CNEA, n◦ 14/2023). The animals were housed at 24 °C with a 12/12 h light/dark cycle, with tap water and a standard diet (Cooperación, Buenos Aires, Argentina) supplied *ad libitum*. Cage changing was performed three times per week. Animal follow-up in terms of tumor development and mucositis after BNCT was performed under intraperitoneal (i.p.) anesthesia [(ketamine (140 mg/kg) and xylazine (21 mg/kg)]. The intravenous injection of Na[*o*-COSAN] solution implied a surgery to expose the jugular vein, under i.p. anesthesia [ketamine (70 mg/kg) and xylazine (10.5 mg/kg)] and i.p. analgesia (Tramadol 6 mg/kg/day, the day before and 48 h after surgery). The same protocol of analgesia was employed when BNCT-treated animals exhibited severe mucositis (until its resolution).

### 4.2. Na[o-COSAN] Solution

For the biodistribution studies, Na[*o*-COSAN] made with natural boron (^11^B) was used. For BNCT studies, Na[*o*-^10^COSAN] enriched with ^10^B was employed. The solutions were made with miliQ water (7.5 mg B/kg of body weight = 0.5 mL Na[*o*-COSAN]/100 g of body weight).

### 4.3. Biodistribution Studies

Hamsters bearing tumors were subjected to Na[*o*-COSAN] biodistribution studies at 7.5 mg B/kg as a slow bolus injection in the jugular vein under ketamine-xylazine anesthesia. The Na[*o*-COSAN] dose was chosen based on previous studies on mice [38]. The hamsters were euthanized at different time-points after injection: (A) 30 min (*n* = 2 animals); (B) 2 h (*n* = 3 animals); (C) 3 h (*n* = 3 animals); (D) 5 h (*n* = 3 animals); (E) 18 h (*n* = 2 animals). Samples of blood, tumor, precancerous tissue, normal pouch tissue, liver, spleen, kidney, and lung were taken in polypropylene tubes pre-weighed (between 30 and 50 mg). Samples were digested with nitric acid for 1 h at 100 °C. Once the digestion was complete, 0.2 mL of the solution yttrium (0.5 ppm) was added as an internal standard prior to the addition of 0.55 mL of a 5% Triton X-100 solution in water. Blood samples (200–300 mL) were digested in 1.25 mL of nitric acid for 1 h at 100 °C. Then, 1 mL of the solution of yttrium (0.5 ppm) was added prior to the addition of 2.75 mL of a 5% Triton X-100 solution in water. Afterwards, digested tissues/organs and blood samples were sonicated at 60 °C for 1 h. Standard solutions of boric acid (enriched to 99.8% ^10^B) were used to prepare a calibration curve each day. Boron measurements were performed with inductively coupled plasma-atomic emission spectroscopy (ICP-OES 5110, Agilent Scientific Instruments, Santa Clara, CA, USA), using the boron 249.677-nm analytical line.

Each hamster had a variable number of tumors. Tumor/blood, tumor/precancerous tissue and tumor/normal tissue boron concentration ratios were calculated for each tumor considering the mean value of the samples corresponding to that particular tumor and the mean blood value, precancerous tissue value, or normal tissue value corresponding to the hamster bearing that particular tumor.

### 4.4. BNCT In Vivo Studies

#### 4.4.1. Irradiation Procedure

Based on the results obtained in our biodistribution studies, tumor-bearing animals were treated with BNCT mediated by Na[*o*-COSAN] at 7.5 mg B/kg b.w., 3 h after injection (*n* = 8 animals). Irradiations were performed at the RA-3 nuclear reactor (Ezeiza, Buenos Aires, Argentina), prescribing 5 Gy absorbed dose to the precancerous tissue around tumors. The precancerous tissue was considered the dose-limiting tissue, based on toxicity data obtained in previous BNCT studies with other boron compounds [29,49]. To protect the body of the animals from thermal neutrons, a ^6^Li carbonate shielding was employed, allowing the cheek-pouch-bearing tumors to be everted out of the enclosure onto a protruding shelf for exposure.

#### 4.4.2. Evaluation of Mucositis

The hamsters were followed weekly, during 4 weeks post-irradiation, assessing clinical signs and body weight. We monitored toxicity induced by BNCT in terms of mucositis in precancerous tissue, employing a 5-grade scale based on mucositis studies in humans and hamsters [49]. Grade 0: healthy appearance, no erosion or vasodilation; Grade 1 (slight): erythema and/or edema and/or vasodilation, no evidence of mucosal erosion; Grade 2 (slight): severe erythema and/or edema, vasodilation and/or superficial erosion; Grade 3 (moderate): severe erythema and/or edema, vasodilation, and the formation of ulcers <2 mm in diameter; Grade 4 (severe): severe erythema and/or edema, vasodilation, and the formation of ulcers ≥2 mm and <4 mm in diameter, and/or necrosis areas <4 mm in diameter; Grade 5 (severe): the formation of ulcers ≥ 4 mm in diameter or multiple ulcers ≥2 mm in diameter, and/or necrosis areas ≥ 4 mm in diameter. Grading was based on the most severe macroscopic feature.

#### 4.4.3. Evaluation of Tumor Response

BNCT therapeutic effect was evaluated as the percentage of tumors with partial remission (PR), complete remission (CR), overall (partial + complete) response (OR), and no response in each tumor volume category at the time of irradiation: small (<10 mm^3^), medium (≥10 <100 mm^3^), and large tumors (≥100 mm^3^). Cancerized, untreated animals served as controls (*n* = 8 animals).

## Figures and Tables

**Table 1:** Boron concentration values (ppm) for different and relevant tissue/organs at 2 h, 3 h, and 5 h after Na[*o*-COSAN] injection. For tumors, (*n*) is the number of samples. For the rest of the tissue/organs, (*n*) is the number of animals.

Time Post Na[*o*-COSAN]						
Boron Concentration (Mean ppm ± SD) (*n*)						
	2 h		3 h		5 h	
Blood	21.4 ± 2.9	(3)	30.5 ± 15.7	(3)	23.5 ± 3.8	(3)
Tumor	11.9 ± 4.0	(11)	13.2 ± 4.7	(8)	10.8 ± 2.9	(17)
Precancerous tissue	7.2 ± 1.2	(3)	7.5 ± 1.0	(3)	6.1 ± 1.1	(3)
Normal pouch tissue	6.8 ± 2.0	(3)	4.8 ± 1.4	(3)	3.2 ± 0.7	(3)
Liver	32.8 ± 5.7	(3)	34.9 ± 2.7	(3)	28.1 ± 5.3	(3)
Spleen	16.6 ± 4.0	(3)	16.7 ± 1.9	(3)	10.9 ± 1.9	(3)
Kidney	18.2 ± 7.3	(3)	12.4 ± 2.7	(3)	18.2 ± 14.3	(3)
Lung	76.0 ± 16.5	(2)	74.3 ± 6.2	(3)	61.9 ± 7.9	(3)

**Table 2:** Boron concentration ratios: tumor/blood (T/B), tumor/precancerous tissue (T/Pr), and tumor/normal pouch tissue (T/N).

		T/B	T/Pr	T/N
**Time Post Na[*o*-COSAN]**	**2 h**	0.6 ± 0.2	1.6 ± 0.1	1.8 ± 0.3
	**3 h**	0.5 ± 0.3	1.8 ± 0.7	2.9 ± 1.5
	**5 h**	0.5 ± 0.2	1.8 ± 0.3	3.4 ± 0.3

**Table 3:** Percentage of hamsters with mucositis at different time-points after BNCT mediated by Na[*o*-^10^COSAN]; (*n*) = 8 animals in each time-point.

Mucositis Grade		Percentage of Hamsters					
		Time (Days)					
		0	7	10	14	21	28
**0**		12.5	0	0	0	0	0
**1**	**slight**	87.5	25	0	37.5	100	100
**2**		0	25	62.5	50	0	0
**3**	**moderate**	0	25	37.5	12.5	0	0
**4**	**severe**	0	25	0	0	0	0
**5**		0	0	0	0	0	0

**Table 4:** Percentage of tumors with partial remission (PR), complete remission (CR), and overall tumor response (OR = PR + CR) as a function of time after Na[*o*-^10^COSAN]/BNCT vs. the control group (cancerized, untreated animals); (*n*) is the number of tumors.

	7 Days				14 Days				21 Days				28 Days			
	(*n*)	%OR	%PR	%CR	(*n*)	%OR	%PR	%CR	(*n*)	%OR	%PR	%CR	(*n*)	%OR	%PR	%CR
Control *	(51)	24	22	2	(51)	22	14	8	(45)	18	11	7	(34)	21	12	9
**Na[*o*-^10^COSAN]/BNCT**	(47)	94	68	26	(47)	94	38	55	(47)	89	45	45	(42)	81	29	52

* Data reported for the control group were taken from Monti Hughes et al. [43]. OR, PR, and CR in this group are due to spontaneous remissions.

**Table 5:** Therapeutic effect of Na[*o*-^10^COSAN]/BNCT considering tumor volume: percentage of tumors with partial remission (PR), complete remission (CR), and overall response (OR = PR + CR) at 28 days after BNCT (end of animal follow-up). (*n*) is the number of tumors.

Tumor Volume Category		Tumor Remission (as a Percentage)		
	(*n*)	PR%	CR%	OR%
**Total**	(42)	29	52	81
**Large ≥ 100 mm^3^**	(8)	75	13	88
**Medium ≥10 <100 mm^3^**	(12)	42	42	83
**Small < 10 mm^3^**	(22)	5	73	77

## Data Availability

The datasets used for the current study are available from the corresponding author on reasonable request.

## Supplementary Materials

The following supporting information can be downloaded at: https://www.mdpi.com/article/10.3390/ph17101367/s1. References [40,41,52] are cited in the supplementary materials.
