# Supplementary material for: Cobaltabis(Dicarbollide) [o-COSAN]− for Boron Neutron Capture Therapy of Head and Neck Cancer: Biodistribution and Irradiation Studies in an Experimental Oral Cancer Model
Source: Pharmaceuticals (Basel). 2024 Oct 14;17(10):1367. doi: 10.3390/ph17101367 (PMC11510372; doi:10.3390/ph17101367)
Supplement: Supplementary file 1 [file pharmaceuticals-17-01367-s001.zip › pharmaceuticals-3222148-supplementary.pdf]

Supplementary Materials:

Experimental section:

Synthesis and characterization of ^{10}B enriched $[HNMe_3][7,8\text{-}C_2{}^{10}B_9H_{12}]$; ^{10}B enriched-Cs[*o*-^{10}COSAN] and ^{10}B enriched Na[*o*-^{10}COSAN].

Experimental Section.

Materials: NaCl was purchased from Sigma–Aldrich, whereas the cationic exchanging resin used (Amberlite IR120, H form) was purchased from Acros Organics and the hydrochloric acid (37%) was purchased from Carlo Erba Reagents. Solvents used were from Carlo Erba SDS and purified by distillation from sodium and benzophenone under a nitrogen atmosphere before use. ^{10}B enriched-Cs[*o*-COSAN], was synthesized from ^{10}B enriched-$[HNMe_3][7,8\text{-}C_2B_9H_{12}]$ by a method [1] that is a modification of the original synthesis by Hawthorne *et al.* [2] ^{10}B enriched-$[HNMe_3][7,8\text{-}C_2B_9H_{12}]$ was obtained from neutral *closo* 1,2-$C_2B_{10}H_{12}$ as described in the literature by Hawthorne. [3] >98% ^{10}B enriched- *closo*-1,2-$C_2B_{10}H_{12}$ was purchased from Katchem Spol.sr.o. $CoCl_2$ was purchased from Sigma Aldrich. ^{10}B enriched-Na[*o*-COSAN] was obtained by the Cation exchange chromatography column method.

Instrumentation and Measurements: The ^{1}H NMR (400.13 MHz) and ^{10}B NMR (42.98 MHz) spectra were recorded on a Bruker Avance NEO 400 instrument equipped with the appropriate decoupling accessories. All NMR spectra were performed in deuterated acetone at 22ºC. The ^{10}B NMR chemical shift values were referenced to external $BF_3{\cdot}OEt_2$, while the ^{1}H NMR chemical shift values were referenced to $SiMe_4$. Chemical shifts are reported in units of parts per million downfield from reference. The mass spectra were recorded in the negative ion mode using a Bruker Biflex MALDI-TOF-MS [N_2 laser; λ_{exc} 337 nm (0.5 ns pulses); voltage ion source 20.00 kV (Uis1) and 17.50 kV (Uis2)].

Synthesis of ^{10}B enriched $[HNMe_3][7,8\text{-}C_2{}^{10}B_9H_{12}]$. 204 mg (1.50 mmol) of >98% ^{10}B enriched- *closo*-1,2-$C_2B_{10}H_{12}$ and 471 mg of KOH (85%) (7.14 mmol) were added to a round-bottom flask with 11 mL of EtOH absolute (synthesis grade). The mixture was refluxed for 5h and brought to pH=7 adding drops of 1M HCl. If necessary, more HCl was added until the precipitate disappeared. EtOH was evaporated and a saturated solution of $NMe_3{\cdot}HCl$ was added for the precipitation of a white solid. It was vacuum filtered and washed with water (3 x 5mL), petroleum ether (3 x 5 mL) and air dried. 244 mg of product were obtained, starting material contained an impurity of 6% $B(OH)_3$, taking that into account the obtained yield was 93.1%. $^{1}H\{^{10}B\}$ NMR (400 MHz, CD_3COCD_3) δ: 3.22 ($HNMe_3^+$), 1.97 (B-H), 1.72 (B-H), 1.70 (C_c-H), 1.29, 1.24, 0.59 and 0.14 (B-H), -2.88 (br s, apical H). $^{10}B\{^{1}H\}$ NMR (42.98 MHz, CD_3COCD_3) δ: -10.9 (d, 2 B), -16.7 (d, 2 B), -17.7(d, 1 B), -22.3(d, 2 B), -33.2(dd, 1 B), -37.9(d, 1 B). MALDI-TOF MS: m/z (%) M of ^{10}B enriched-[7,8-

$C_2B_9H_{12}]^-$ theoretical 126.23, experimental m/z=126.15 [M, 100%]. FTIR: $HNMe_3^+$:3148 cm^{-1}, ν(C_c-H): 3026 and 2744 cm^{-1}, ν(B-H): 2514 cm^{-1}.

Synthesis of ^{10}B enriched Cs[*o*-^{10}COSAN]. 200 mg (1.58 mmol) of ^{10}B enriched-[$HNMe_3$][7,8-$C_2B_9H_{12}$] were added to an Schlenk flask and degassed. The flask was placed in a 0 ºC bath and 10 mL of dry THF and 1.68 mL of 1.6 M solution of tBuOK (2.69 mmol) in dry THF were added. The mixture was stirred, after 1h the ice bath was removed and left stirring for an extra hour. The white mixture was cannuled into a suspension of 243 mg of previously degassed anhydrous $CoCl_2$ (1.87 mmol) in 10 mL of dry THF in inert atmosphere, the black solution was refluxed for 2h in inert conditions. After cooling, the crude was paper filtered to remove metallic cobalt and rota vapored. The remaining solid was dissolved in 25 mL of Et_2O and extracted with 3x 10 mL of 0.1M HCl. The aqueous phases were extracted with 25 mL of Et_2O and all organic phases were collected, dried with $MgSO_4$ and evaporated. The solid was dissolved in the minimum amount of distilled water and a saturated aqueous solution of CsCl was added for the precipitation of an orange solid which was vacuum filtered, washed with water (3 x 5mL), petroleum ether (3 x 5 mL) and air dried. 182 mg of product were obtained (yield: 74.5%). ^{1}H{^{10}B} NMR (400 MHz, CD_3COCD_3) δ: 3.96 (C_c-H), 3.41 (B-H 8,8′), 3.00 (B-H 10,10′), 2.73 (B-H 4,4′,7,7′), 1.96 (B-H 9,9′,12,12′), 1.63 (B-H 6,6′), 1.58 (B-H 5,5′,11,11′). ^{10}B{^{1}H} NMR (42.98 MHz, CD_3COCD_3) δ: 6.4 (B 8,8′), 1.17 (B 10,10′), -5.5 (4,4′,7,7′), -6.3 (B 9,9′,12,12′), -17.5 (B 5,5′,11,11′), -23,0 (B 6,6′). MALDI-TOF MS: m/z (%) Molecular weight (M) of ^{10}B enriched-[*o*-^{10}COSAN]$^-$: theoretical 309.38, experimental m/z=309.086 [M, 88.28%], 310.090 [M containing one ^{11}B atom, 11.71%]. Sample of ^{10}B enriched Cs[*o*-^{10}COSAN] contains 99.35% of ^{10}B. FTIR: ν(C_c-H): 3041, 2921 and 2856 cm^{-1}, ν(B-H): 2538 cm^{-1}.

Preparation of ^{10}B enriched Na[*o*-^{10}COSAN]. 90 mg of ^{10}B enriched-Cs[*o*-COSAN] were dissolved in 2 ml of distilled water and 2 ml of acetonitrile. The solution was passed through a Na^+ loaded Cation exchange chromatography column 10 times. The column was water washed until the orange color disappeared and all the mobile phase was rotavaped, the humid solid was vacuum dried at 40-50 ºC for 1h. 73.5 mg of water-soluble solid were obtained (96% yield, Na^+ coordinates 2.5 H_2O). ^{1}H{^{10}B} NMR (400 MHz, CD_3COCD_3) δ: 3.95 (C_c-H), 3.39 (B-H 8,8′), 2.98 (B-H 10,10′), 2.71 (B-H 4,4′,7,7′), 1.94 (B-H 9,9′,12,12′), 1.62 (B-H 6,6′), 1.57 (B-H 5,5′,11,11′). ^{10}B{^{1}H} NMR (42.98 MHz, CD_3COCD_3) δ: 6.4 (B 8,8′), 1.2 (B 10,10′), -5.5 (4,4′,7,7′), -6.3 (B 9,9′,12,12′), -17.5 (B 5,5′,11,11′), -23.0 (B 6,6′). FTIR: ν_s(H-O-H): 3590 cm^{-1}, ν(C_c-H): 3045 cm^{-1}, ν(B-H): 2579 and 2519 cm^{-1}, ν_s(O-H): 1602 cm^{-1}.

Yield comparison between ^{10}B enriched starting material and natural boron. Reactions were carried out using the same methods but starting with natural B *o*-carborane, reaching a 10% yield

improvement in the case of ^{10}B enriched Cs[*o*-COSAN] *vs.* Cs[*o*-COSAN], as shown in Table S1.

Table S1. Yield comparison between ^{10}B enriched starting material and natural boron.

Product synthesis	Natural B	^{10}B enriched products
[$HNMe_3$][7,8-$C_2B_9H_{12}$]	91.90%	93.10%
Cs[*o*-COSAN]	64.30%	74.50%

Figure S1. ^{1}H-NMR spectrum of ^{10}B enriched $[HNMe_3][7,8\text{-}C_2{}^{10}B_9H_{12}]$.

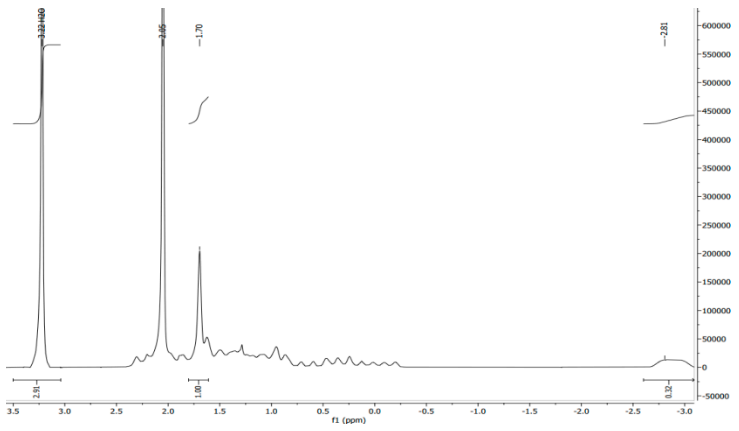

Figure S2. $^{1}H\{^{10}B\}$-NMR spectrum of ^{10}B enriched $[HNMe_3][7,8\text{-}C_2{}^{10}B_9H_{12}]$.

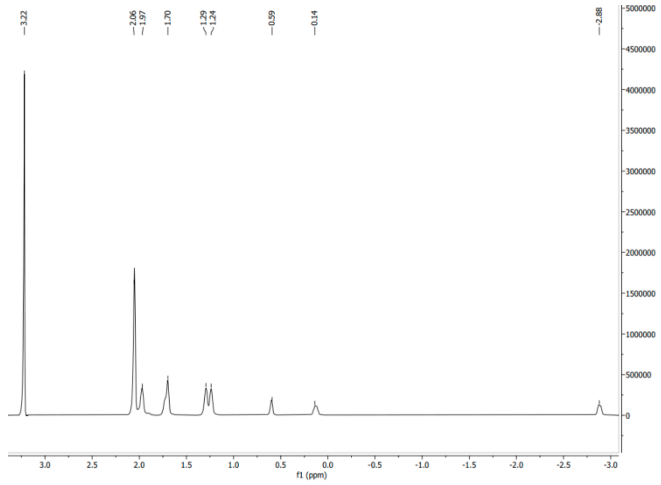

Figure S3. ^{10}B-NMR spectrum of ^{10}B enriched $[HNMe_3][7,8\text{-}C_2{}^{10}B_9H_{12}]$.

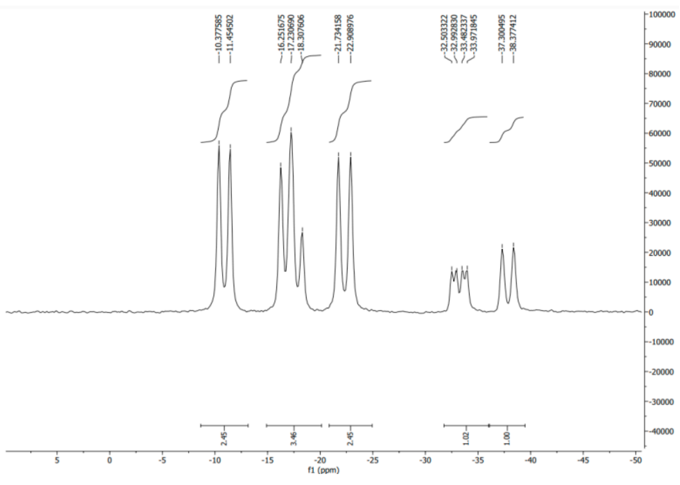

Figure S4. $^{10}B\{^{1}H\}$-NMR spectrum of ^{10}B enriched $[HNMe_3][7,8\text{-}C_2{}^{10}B_9H_{12}]$.

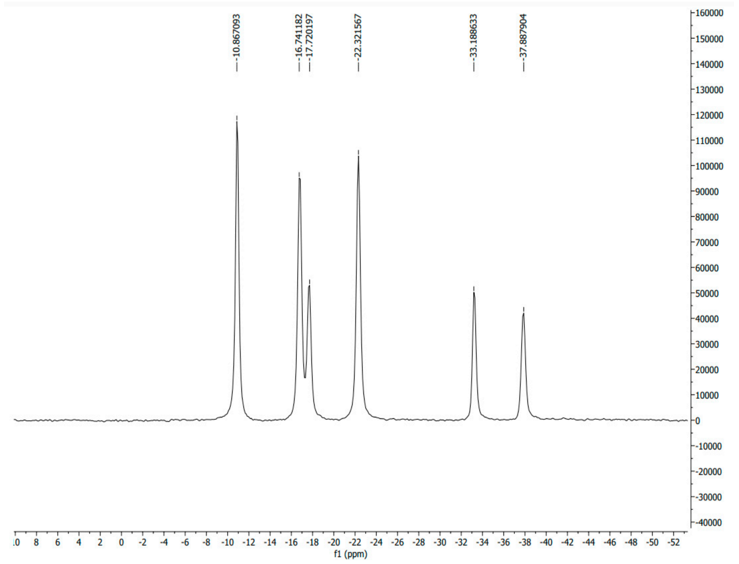

Figure S5. FTIR spectrum of ^{10}B enriched $[HNMe_3][7,8\text{-}C_2{}^{10}B_9H_{12}]$.

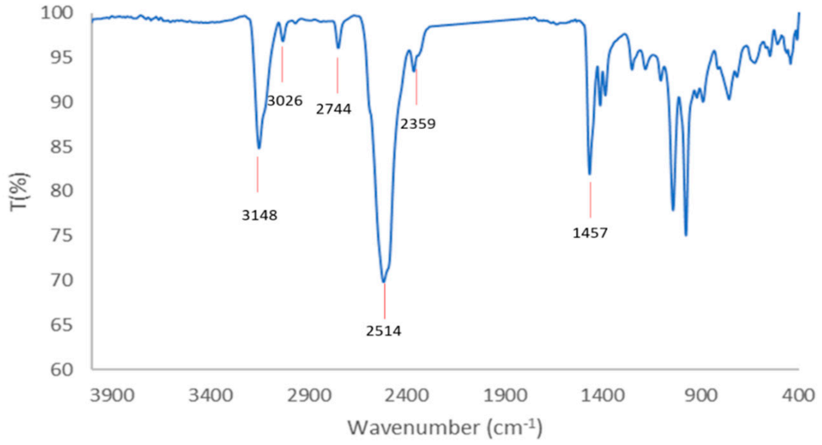

Figure S6. MALDI-TOF spectrum of ^{10}B enriched-$[HNMe_3][7,8\text{-}C_2{}^{10}B_9H_{12}]$.

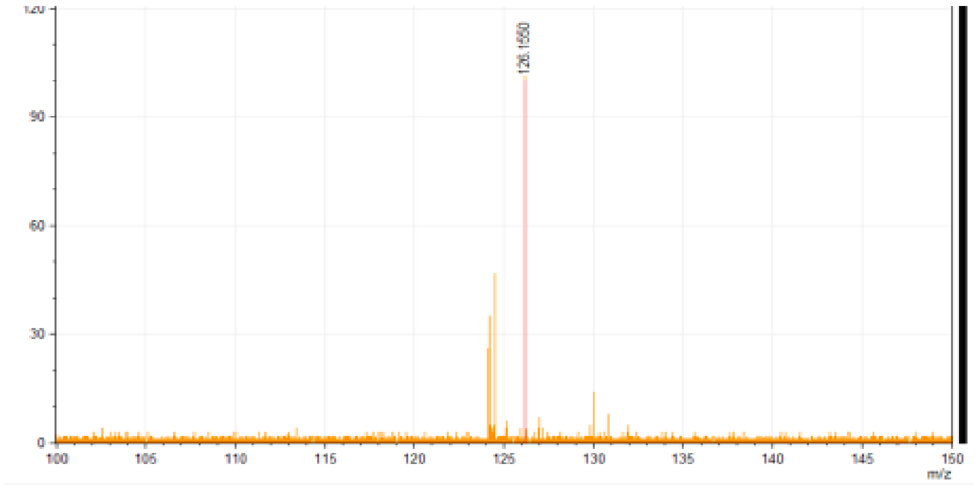

Figure S7. ^{1}H-NMR spectrum of ^{10}B enriched Cs[*o*-^{10}COSAN].

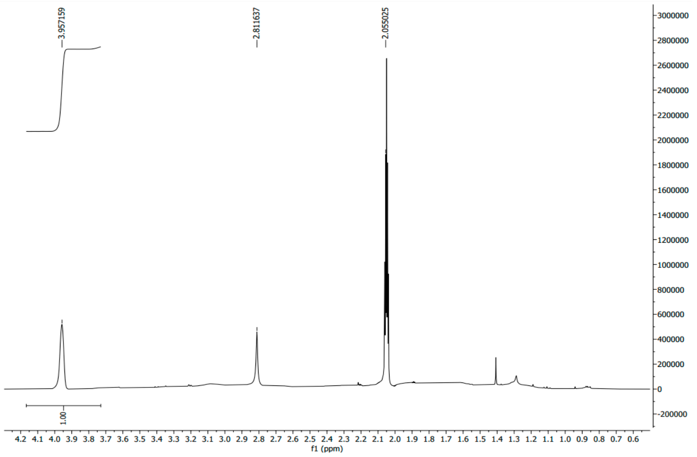

Figure S8. ^{1}H{^{10}B}-NMR spectrum of ^{10}B enriched Cs[*o*-^{10}COSAN].

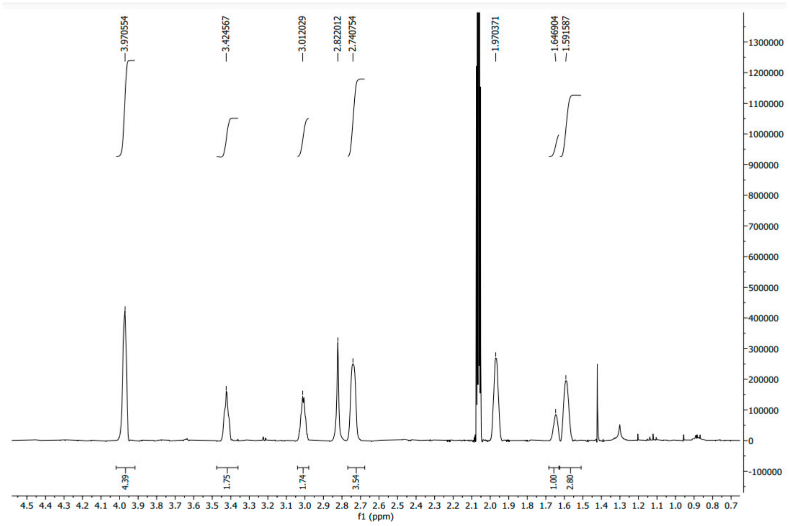

Figure S9. ^{10}B-NMR spectrum of ^{10}B enriched Cs[*o*-^{10}COSAN].

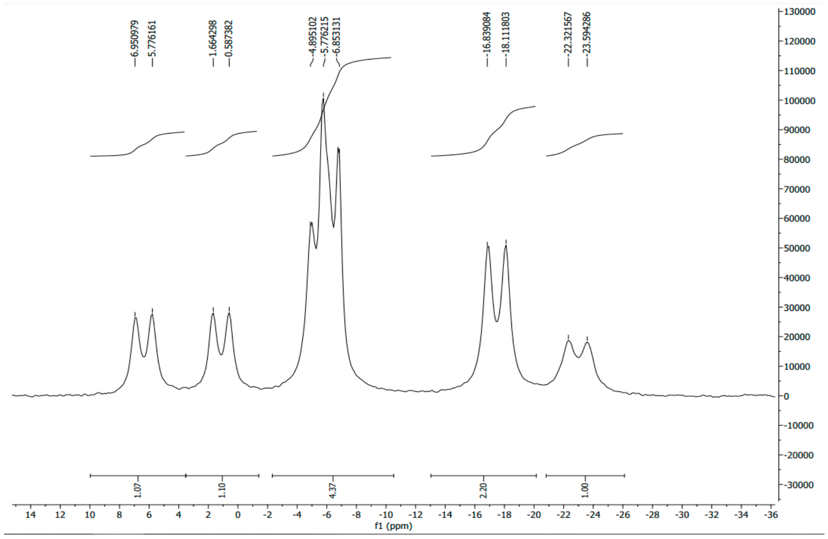

Figure S10. $^{10}B\{^{1}H\}$-NMR spectrum of ^{10}B enriched Cs[*o*-^{10}COSAN].

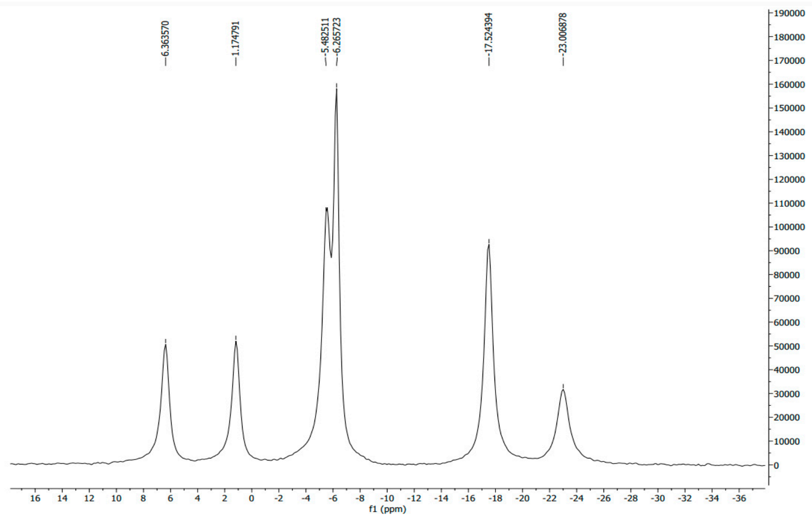

Figure S11. FTIR spectrum of ^{10}B enriched Cs[*o*-^{10}COSAN].

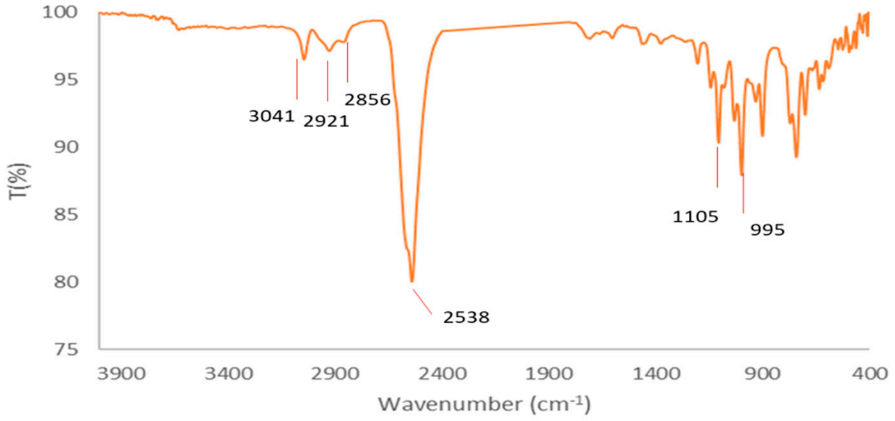

Figure S12. At the top, MALDI-TOF spectrum of ^{10}B enriched Cs[*o*-^{10}COSAN]. At the bottom: MALDI-TOF spectrum of ^{10}B enriched Cs[*o*-^{10}COSAN] (in blue) overlapped with Cs[*o*-COSAN] (in green).

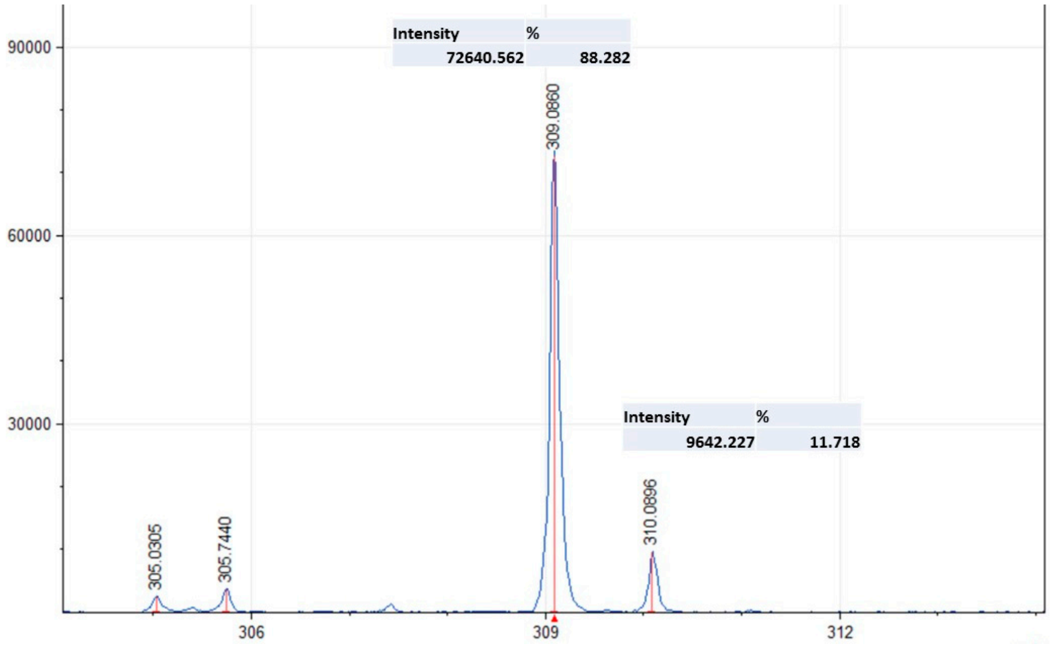

CALCULATION of the percentage of [*o*-^{10}COSAN]$^{-}$ pure in the synthesized ^{10}B enriched Cs[*o*-^{10}COSAN]:

72640.562 of [*o*-^{10}COSAN]$^{-}$ pure 100
9642.227 of [*o*-^{10}COSAN]$^{-}$ with one ^{11}B atom x

964222.7 = 72640.562 x x = 964222.7 / 72640.562 = 13.27

Total number of ^{10}B atoms = 100 x 18 + 13.27 x 17 = 1800 + 225.6 = 2025.6
Total number of ^{11}B atoms = 13.27
Total number of B atoms = 2025.6 + 13.27 = 2038.9

PERCENTAGE of ^{10}B = (2025.6 / 2038.9) x 100 = 99.35%.

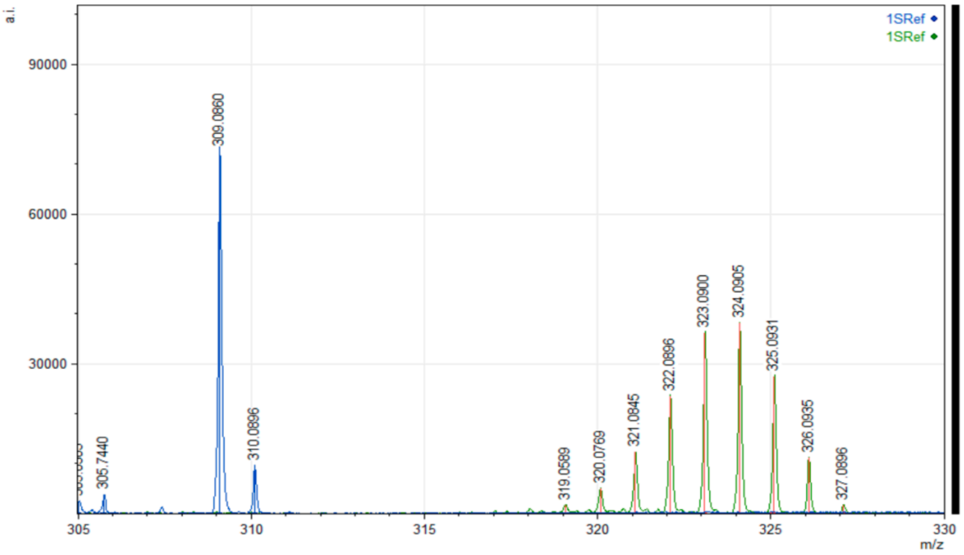

Figure S13. ^{1}H-NMR spectrum of ^{10}B enriched Na[*o*-^{10}COSAN].

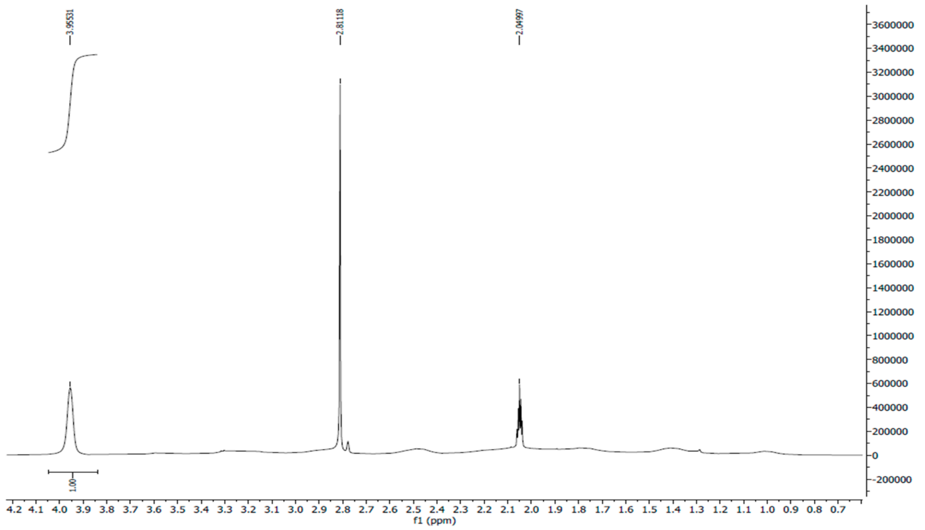

Figure S14. $^{1}H\{^{10}B\}$-NMR spectrum of ^{10}B enriched Na[*o*-^{10}COSAN].

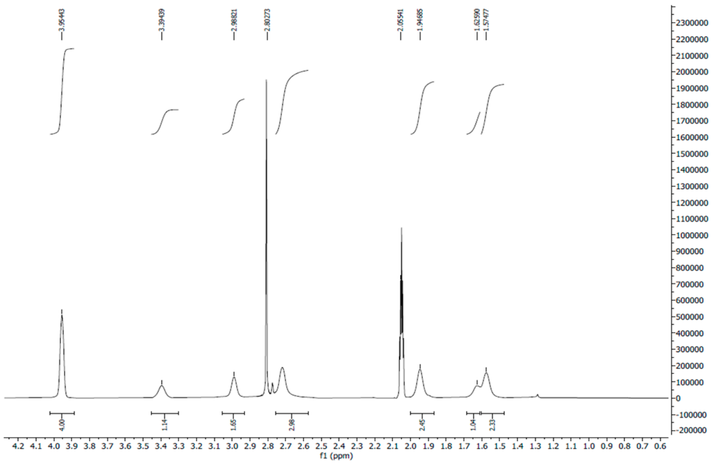

Figure S15. ^{10}B-NMR spectrum of ^{10}B enriched Na[*o*-^{10}COSAN].

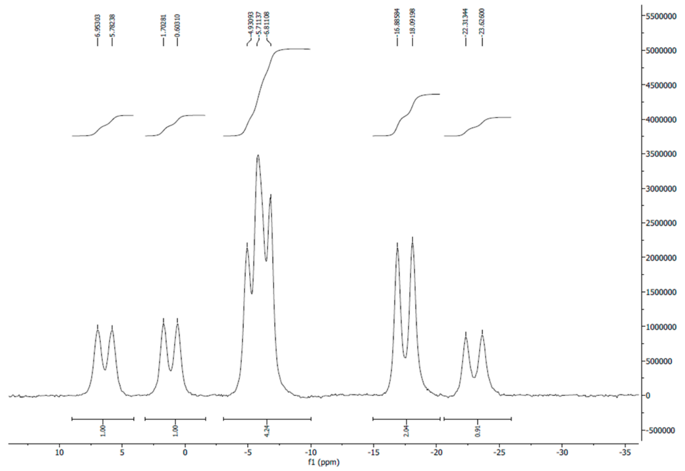

Figure S16. $^{10}B\{^{1}H\}$-NMR spectrum of ^{10}B enriched Na[*o*-^{10}COSAN].

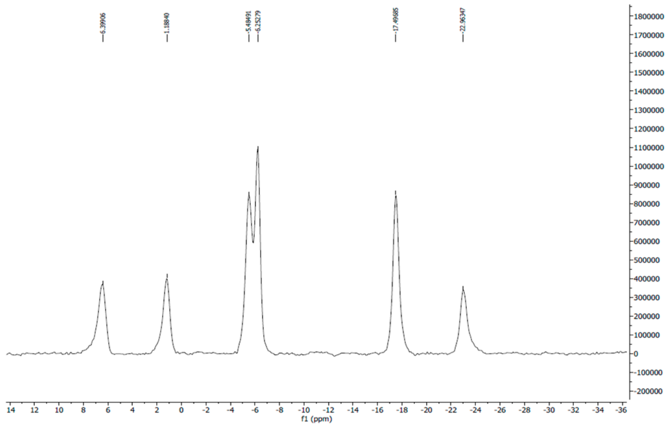

Figure S17. $^{1}H\{^{10}B\}$-NMR spectra of ^{10}B enriched Na[*o*-COSAN] (in grey colour) and ^{10}B enriched Cs[*o*-COSAN] (in orange colour).

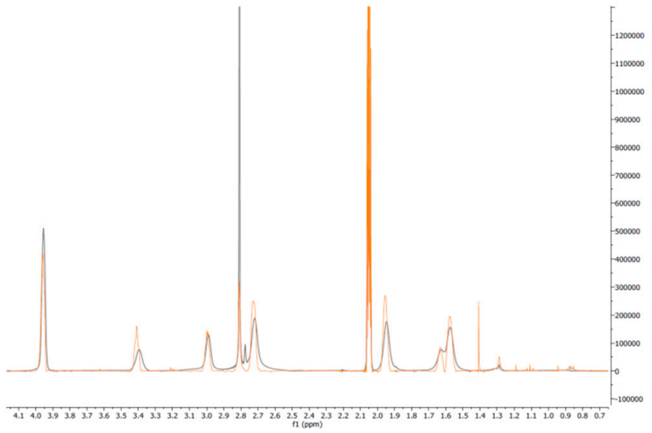

Figure S18. FTIR spectrum of ^{10}B enriched Na[*o*-^{10}COSAN].

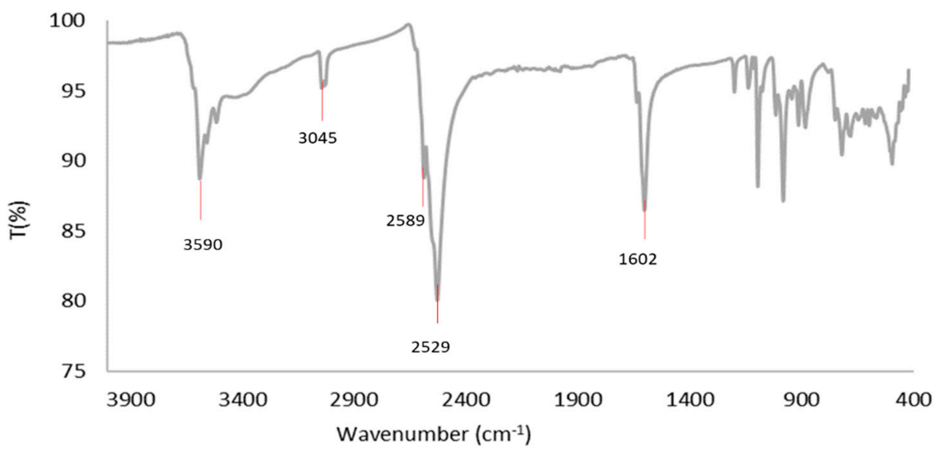

Figure S19. FTIR spectra of ^{10}B enriched Na[*o*-^{10}COSAN] (in grey colour) and ^{10}B enriched Cs[*o*-^{10}COSAN] (in orange colour).

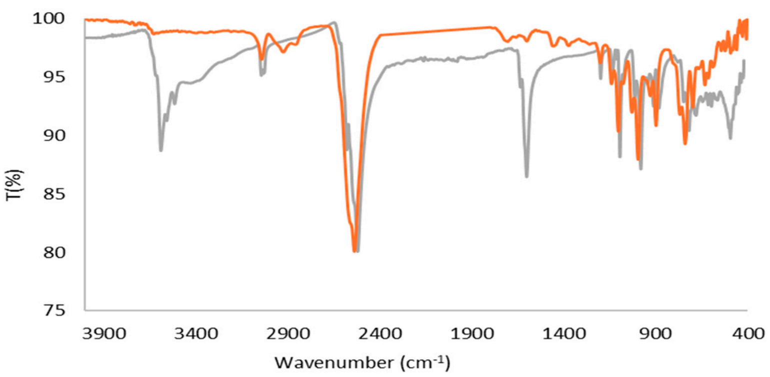

Figure S20. At the top: FTIR spectra of ^{10}B enriched Na[*o*-^{10}COSAN] (in orange colour) and natural boron Na[*o*-COSAN] (in grey colour). At the bottom: The IR spectra in the range 2800-2300 cm^{-1}.

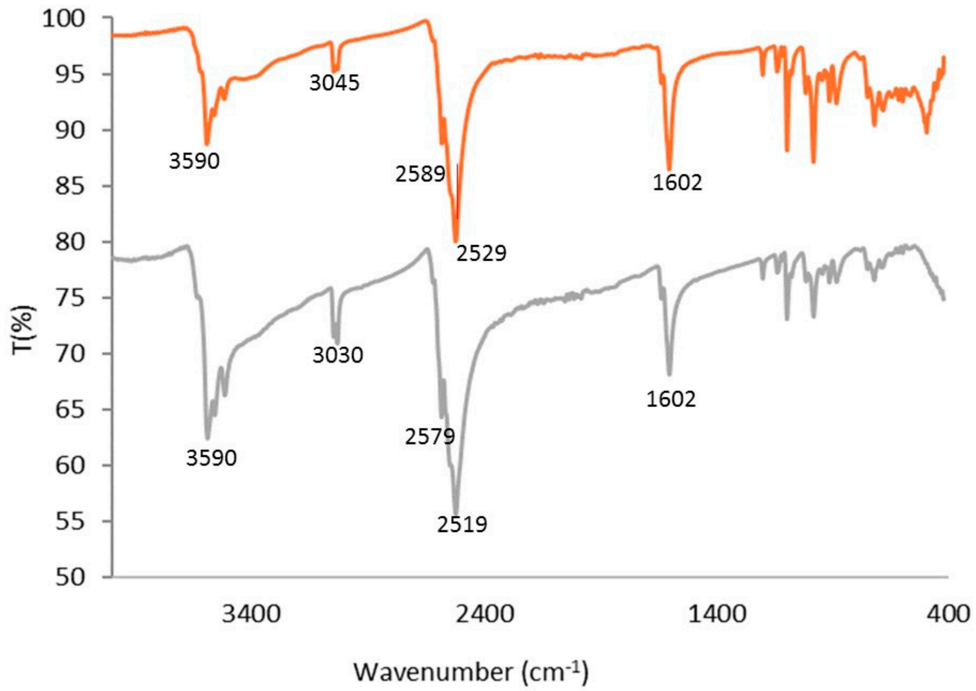

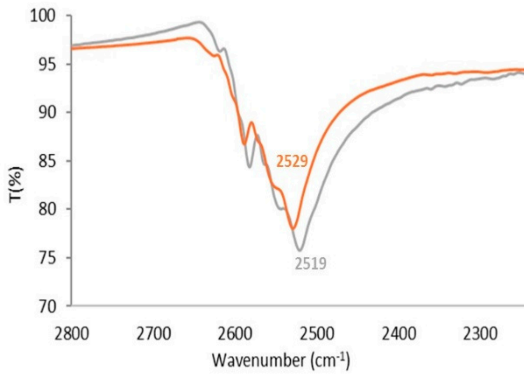

References:

1. C. Viñas, J. Pedrajas, J. Bertran, F. Teixidor, R. Kivekäs, R. Sillanpää, *Inorg. Chem*. **1997**, *36*, 2482–2486.
2. M. F. Hawthorne, D. C. Young, T. D. Andrews, D. V Howe, R. L. Pilling, A. D. Pitts, M. Reintjes, L. F. Warren, P. A. Wegner, *J. Am. Chem. Soc.* **1968**, *90*, 879–896.
3. M. F. Hawthorne, D. C. Young, P. M. Garrett, D. A. Owen, S. G. Schwerin, F. N. Tebbe, P. A. Wegner, *J. Am. Chem. Soc*. **1968**, *90*, 862–868.
